# Participation in the Chronic Phase after Traumatic Brain Injury: Variations and Key Predictors

**DOI:** 10.3390/jcm12175584

**Published:** 2023-08-27

**Authors:** Solveig L. Hauger, Ida M. H. Borgen, Marit V. Forslund, Ingerid Kleffelgård, Nada Andelic, Marianne Løvstad, Paul B. Perrin, Cecilie Røe, Silje C. R. Fure

**Affiliations:** 1 Department of Research, Sunnaas Rehabilitation Hospital, 1453 Bjørnemyr, Norway; marianne.lovstad@sunnaas.no; 2 Department of Psychology, Faculty of Social Sciences, University of Oslo, 0316 Oslo, Norway; 3 Department of Physical Medicine and Rehabilitation, Oslo University Hospital, 0424 Oslo, Norway; idmbor@ous-hf.no (I.M.H.B.); mavfor@ous-hf.no (M.V.F.); uxinff@ous-hf.no (I.K.); nada.andelic@medisin.uio.no (N.A.); cecilie.roe@medisin.uio.no (C.R.); siljfu@ous-hf.no (S.C.R.F.); 4 Center for Habilitation and Rehabilitation Models and Services (CHARM), Institute of Health and Society, Faculty of Medicine, University of Oslo, 0316 Oslo, Norway; 5 Department of Psychology, School of Data Science, University of Virginia, Charlottesville, VA 22904, USA; paul.b.perrin@gmail.com; 6 Central Virginia Veterans Affairs Health Care System, Richmond, VA 23249, USA; 7 Institute of Clinical Medicine, Faculty of Medicine, University of Oslo, 0316 Oslo, Norway

**Keywords:** traumatic brain injury, chronic phase, mild-to-severe TBI, prediction, participation, outcome, rehabilitation, covid

## Abstract

Participation is of major importance for individuals with traumatic brain injury (TBI). This study evaluates participation over a period of one year among persons with TBI in the chronic phase and explores sociodemographic, psychological, and environmental predictors of levels and trajectories of participation. One hundred and twenty home-living survivors of TBI with persistent injury-related consequences at least two years post-injury who participated in a goal-oriented randomized trial were assessed at baseline and after four and twelve months. Linear mixed-effects model analysis was applied to evaluate height, trajectory slope, and predictors of the Participation Assessment with the Recombined Tools-Objective (PART-O) total score and the subscales Productivity, Social Relations, and Being Out and About. Being married, having a higher education, and having good global functioning predicted more frequent participation. Education, executive- and global functions predicted Productivity, while age and being married predicted Social Relations. Participating in the study during the COVID-19 pandemic had a negative impact on Productivity. Participation was relatively stable over 12 months, with a slight decline, but may be influenced by demographic factors and functional consequences. Rehabilitation services should particularly focus on people with TBI living alone with lower levels of global and executive function.

## 1. Introduction

Traumatic brain injury (TBI) represents a substantial global health problem and is considered a chronic disease with a potentially life-long impact on health and well-being [1,2]. The long-term consequences of TBI commonly involve a wide range of symptoms that relate to physical, emotional, cognitive, behavioral, and psychosocial functioning, often resulting in problems with community integration and productivity [2,3,4].

Community reintegration and return to productivity are aspects of participation considered to be important goals of TBI rehabilitation. The World Health Organization International Classification of Functioning, Disability, and Health (ICF) defines participation as “involvement in a life situation” and conceptualizes participation as one of the key health components [5] and a meaningful target for rehabilitation outcomes. Measuring participation after rehabilitation is also a challenge given the broad spectrum of overlapping aspects, such as education, work, community integration, social interaction and relationships, communication, transportation, domestic tasks, and a sense of belonging and being included [6]. Commonly used measures of community integration and return to work and school may target important aspects of participation, but more comprehensive tools like the 17-item Participation Assessment with Recombined Tools-Objective (PART-O) [7] have also been developed. The PART-O provides a broad index of participation and is included in the recommendations for Common Data Elements by the National Institute of Neurological Disorders and Stroke for assessing outcomes in social role participation and social competence in the TBI population [8].

Although participation is considered a main target of rehabilitation, participation nonetheless typically remains reduced in individuals with chronic TBI, likely as it is multi-factorially determined [9,10,11,12]. Multiple variables have been shown to be associated with participation outcomes post-TBI, including injury-related and demographic variables, as well as psychological factors. Predictors of participation include higher education, better current cognitive functioning, longer time since injury, shorter duration of posttraumatic amnesia, younger age, as well as resilience, absence of depression, and living with others [10,13,14]. Moreover, a dose-response relationship has been found between the level of depression one-year post-TBI and decreased social participation [15]. Despite increased knowledge of factors associated with participation after TBI, the amount of variance accounted for by these predictive models has generally been 32–38% [10,14].

To date, there are few longitudinal studies of participation following TBI as assessed by PART-O. A longitudinal study by Hart and colleagues [16] examined patterns of change in social participation among 375 individuals between one and two years after moderate-to-severe TBI. They found that for most individuals, social participation remained stable, but for 25% of participants, an equal proportion either declined or improved in social participation over one year. Improvement was predicted by private insurance status, and decline was predicted by a reduction in functional outcome from year one to year two post-injury. They also found a marginal association between higher education and improved social participation, and that being single at year one marginally predicted a decline in social participation [16]. There is still a need for studies investigating patterns of change in participation among individuals living with the long-term consequences of TBI.

A randomized controlled trial (RCT) evaluating the effect of an individualized goal-oriented intervention in the chronic phase of TBI [17] did not find between-group effects in social participation, as measured by the Social Relations subscale from PART-O. However, the study showed significantly improved generic health-related quality of life as well as reduced self-reported TBI- and anxiety-related symptom levels in the intervention group compared with the control group [17]. The trial included 120 persons with TBI with verified intracranial lesions and persistent symptoms at least two years since injury (time of inclusion) and was partly undertaken during the COVID-19 pandemic. Although no group differences were found, the control group revealed a significant decrease in social participation over the one-year study period. Hence, although the physical and cognitive impairments after TBI are assumed to be relatively stable in the chronic phase (>two years after injury), societal involvement may change. The main aim of the current study was to evaluate participation over a period of one year among the TBI survivors taking part in the above-mentioned goal-oriented RCT. Participation levels and trajectories were evaluated by using self-reported participation levels measured by the PART-O total score and the subscales of Productivity, Social Relations, and Being Out and About. A second aim was to explore sociodemographic, psychological, and environmental predictors of participation level and trajectory slope. We hypothesized that the level of participation and trajectory slope over one year in the chronic phase following TBI would be associated with factors related to sociodemographics, such as age, sex, living with a partner, and level of education, as well as related to TBI-related symptoms, such as cognitive deficits, emotional symptoms, and level of global functioning. We also hypothesized that study enrollment and follow-up during the COVID-19 pandemic with social restrictions would be associated with a reduction in participation among study participants.

## 2. Materials and Methods

### 2.1. Study Design

This study includes a sample of participants from an RCT with a one-year follow-up (NCT03545594 https://clinicaltrials.gov accessed on 24 August 2023). One hundred and twenty persons were included and randomized to either an intervention (i.e., receiving a home-delivered individualized goal-oriented rehabilitation program, *n* = 60) or a control group (i.e., receiving treatment as usual, *n* = 60) [18]. As no significant between-group difference was found regarding participation level at the one-year follow-up for the Social Relation subscale of the PART-O, the sample was analyzed as one merged cohort in the current study, while also controlling for group effects for the PART-O total score and the remaining subscales. Data were collected at inclusion, at four to five, and at twelve months after baseline.

### 2.2. Setting

Eligible participants were invited to a baseline assessment (T1) at the TBI outpatient clinic at Oslo University Hospital. If they met inclusion criteria and provided written informed consent, participants were enrolled and randomized. The intervention was delivered in the participant’s home by videoconference or telephone. Outcomes were assessed at the end of treatment at four to five months (T2) and at the one-year follow-up (T3), either at the TBI outpatient clinic or through a combination of phone interviews and mailed questionnaires.

### 2.3. Participants

**Figure 1:**
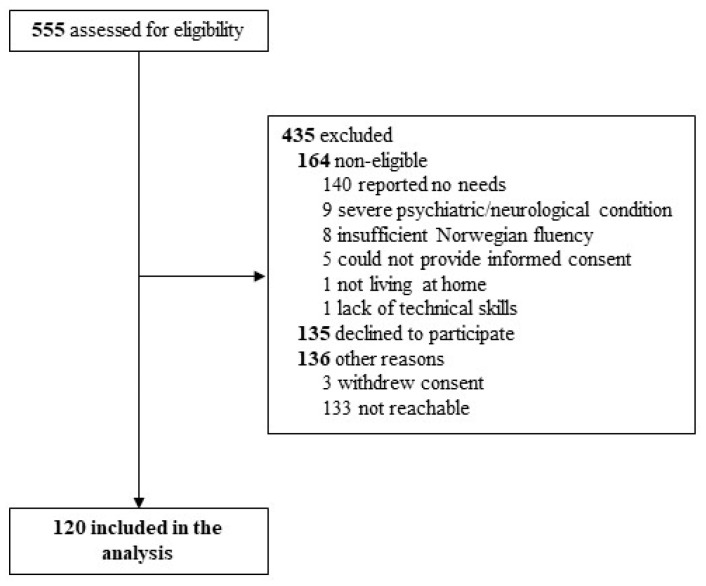
Flowchart of inclusion.

Participants were eligible for study participation if they had been admitted to the trauma referral center in Southeast Norway at Oslo University Hospital with a TBI diagnosis and CT/MRI-verified traumatic intracranial abnormalities. Participants had to be between 18 and 72 years old at the time of inclusion. They had to be in the chronic phase, i.e., at least two years post-injury, and living at home to be considered eligible for inclusion (Figure 1). Included participants needed to report ongoing TBI-related cognitive, emotional, or physical problems or reduced physical and mental health or difficulties with participation in activities with family, friends, or in the community (based on interviews and standardized questionnaires at baseline). Participants were excluded if they had severe progressive neurologic disorders or severe psychiatric disorders that would confound outcome assessments and if they were unable to provide informed consent or participate in a goal-setting process. Participants with insufficient fluency in Norwegian to allow for verbal and written communication with therapists and outcome assessors were also excluded. The recruitment of participants took place between June 2018 and December 2020. The Norwegian government initiated the COVID-19 societal lockdown in Norway on the 12th of March 2020, and the pandemic period in the present study is defined from this date and throughout the study. From this date on, participants lacking the technical skills or equipment to receive videoconferences were excluded. Eligible participants were sent a written invitation to participate, whereafter they were screened by phone. If deemed eligible, they were invited to the baseline assessment. A population-based sample of 555 individuals was invited to participate; only 3 patients withdrew their consent after inclusion, for a total of 120 included in the study sample.

### 2.4. Outcome and Predictor Variables

The main outcome variable was the Norwegian version of the self-reported PART-O, a tool developed to measure participation outcomes in the TBI population [7,19]. The tool is based on the International Classification of Functioning, Disability, and Health (ICF) and targets the long-term participation challenges specific to the TBI population [7,19]. The PART-O has three subscales: Productivity—time spent working, at school, or on homemaking activities; Social Relations—time spent with friends, giving emotional support, and internet communication; and Out and About—time spent outside the home for leisure, shopping, or other purposes. In the original 17-item version, each item is scored from zero to five according to the frequency of activities, with higher scores representing increased participation. Subscale scores are calculated by taking the average of completed items. The measurement properties of the original English version have been validated and refined [20]. The distribution of items and the unidimensionality of the Norwegian PART-O were checked. This showed that the PART-O total as well as the three subscales were unidimensional; however, the Out and About subscale revealed floor effects.

Predictor variables were selected based on clinical experience and previous research [10,14,15,16,21] and were collected at baseline through questionnaires and interviews with participants. The demographic and TBI-related variables included in the analyses were: age (years), sex (male/female), education (years), relationship status (married/cohabiting or single/living alone), work status (employed at baseline yes/no), along with TBI-related functional decline measured by the Glasgow Outcome Scale Extended; GOSE [22,23]. GOSE measures global outcomes after TBI and consists of eight outcome categories, from 1 (dead) to 8 (full functional recovery). Based on the inspection of category frequency in the current sample, the categories were regrouped into ≤5 = severe or lower moderate disability, 6 = upper moderate disability, and 7 = lower good recovery, while category 8 was omitted in the analysis as none of the included participants scored as fully recovered at baseline.

Depression was assessed using the Patient Health Questionnaire-9 (PHQ-9) [24]. The PHQ-9 is a self-report measure of depression symptom severity that uses a frequency scale of 0 (not at all) to 3 (nearly every day) for each of the nine Diagnostic and Statistical Manual of Mental Disorders (Fourth Edition) [25] symptoms of depression during a time frame of the previous two weeks. The item scores are summed for the total score and dichotomized at <10 or ≥10 for analytic purposes, with a score ≥ 10 representing clinically significant depressive symptoms.

TBI-related symptoms were measured by the Rivermead Post-Concussion Symptoms Questionnaire (RPQ) [26]. Participants reported symptoms over the last week compared with before the TBI. In total, 16 items are scored on a Likert scale from 0 to 4 (no problem to severe problem). The average score is calculated by omitting score 1 (i.e., no longer the presence of a symptom).

Self-reported cognitive functioning was measured using the Behavior Rating Inventory of Executive Function—Adult version (BRIEF-A). The BRIEF-A is a standardized questionnaire for measuring self-reported executive functions in everyday life [27]. Statements are answered on a three-point Likert scale ranging from 1 (never) to 3 (often), reflecting executive difficulties during the past six months. Based on nine subscales, the BRIEF-A provides an overall Global Executive Composite (GEC) score.

Study participation during the COVID pandemic was measured through a COVID variable with four categories: 0 (subjects had all assessments before the pandemic), 1 (baseline and four-months assessment before, and only the 12-months assessment during the pandemic), 2 (baseline before the pandemic, and both four-months and 12-months assessments during the pandemic), and 3 (all assessments during the pandemic).

### 2.5. Statistical Analysis

Data analyses were performed with Stata 17. Descriptive statistics were used to describe socio-demographic- and injury-related variables, and results are presented as percentages and means with standard deviations (SDs) or in the median with 1st and 3rd quartiles (Q1 and Q3). The independent variables included sociodemographic (age, sex, relationship status, level of education, work status), TBI-related and emotional symptoms (TBI-related symptom burden, depression, anxiety), functioning (cognitive functioning, global functioning), and participation during the COVID-19 pandemic. Any correlation (Spearman’s rho) between the independent variables and PART-O and its subscales was checked with a cutoff of >0.7. Hence, employment at baseline was omitted due to the high correlation with GOSE, and anxiety was omitted due to the high correlation with depression. Linear mixed-effects model analysis was applied in four different models to predict the PART-O total score and the three subscales of PART-O. The only missing value at baseline was BRIEF-A for one participant, which was imputed using the mean value of BRIEF-A of the other participants from the same GOSE category. Three participants did not attend follow-ups at T2 and T3, in addition to five participants having missing values for PART-O. The missing values represented only 6.7% of the data and were handled by the maximum likelihood estimations of the model. Group allocation (time-by-treatment group interaction) and participation during the COVID-19 pandemic were controlled for in the analysis. Potential predictors were analyzed as fixed effects, allowing a random intercept and random effect of time. The significance level was set at 0.05.

## 3. Results

**Figure 2:**
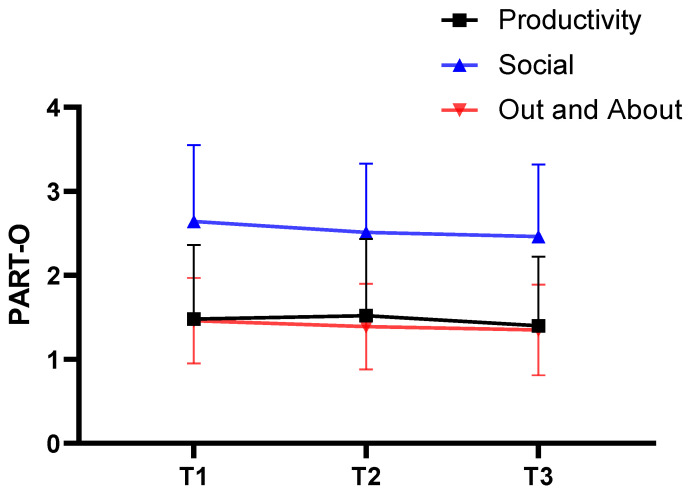
Mean values for PART-O Productivity, Social, and Out and About subscales for the total sample of patients with TBI at baseline (T1), four months (T2), and 12 months (T3) follow-up.

Baseline characteristics of the sample are presented in Table 1, showing a representative sample of mild to severe TBI with the typical predominance of males (71%). Participation levels were higher on the Social Relation subscale compared with the Productivity and Out and About subscales (Figure 2). Regarding study participation during the COVID-19 pandemic, 26 (22%) of the participants had all assessments (T1, T2, and T3) conducted before the pandemic started, while 34 (28%) went through all assessments during the pandemic. For 28 (23%) of the participants, only the 12-month (T3) follow-up took place during the pandemic, and 32 (27%) had both four- and 12-month (T2 and T3) follow-ups taking place during the pandemic.

The PART-O total score was 1.86 at baseline, 1.82 at 4 months, and 1.74 at 12 months. The model showed a small, but significant, negative effect of time on the PART-O total score (coeff. −0.02, *p* = 0.014, 95% CI −0.03 to −0.01), with a decrease in participation over time. No significant effect of time was found for any of the PART-O subscales (Table 2), (Figure 2).

Positive predictors of participation as measured by higher PART-O total scores were younger age, being in a relationship, having higher education, and having higher global functioning, while longer trajectory time predicted lower participation (Table 2). Having higher education and higher global (GOSE) and self-reported executive cognitive (BRIEF-A) functioning predicted better scores on the PART-O Productivity subscale while having assessments during the COVID-19 pandemic had a negative impact on Productivity. Younger age and being in a relationship predicted higher PART-O Social Relation scores (Table 2).

The model was non-significant for the Out and About subscale, (*p* = 0.164) with no significant predictors, and subsequently, the model for the Out and About subscale was not found valid for the identification of predictors.

## 4. Discussion

The present study indicates a very slight, but significant, decline in participation in the chronic phase after TBI as measured by PART-O and its subscales of Productivity and Social Relation. The Out and About subscale did not fit the model and could not be assessed separately. Possibly, the observed floor effects in the Out and About subscale contribute to the latter. Relatively stable levels of social participation have also previously been found between one and two years following moderate-to-severe TBI [16].

Community integration, a construct associated with participation, has also been found to improve significantly during the first year post-injury [20,28], and, importantly, in the very late phase from 10 to 20 years post-injury [4]. In the current study, all participants were in the chronic phase of TBI; however, the sample had a large heterogeneity with regards to time since injury, with a median of 4 years (range 2–24). Hence, the small decline in participation levels is not likely explained by time-specific milestones in the course of injury, such as transition to the community and return to work. As the minimal clinically important difference of PART-O is not established, the clinical relevance of the small decline in participation is undetermined in the current study.

Partaking in the study during the COVID-19 pandemic did not significantly affect the mean PART-O total score at a group level but had a negative impact on the Productivity subscale. Such influence has been documented in a previous longitudinal study of individuals with TBI during and after the initial lockdown phase of COVID-19 [29]. As this study was undertaken from 2018 to 2021, participants were differentially affected by the pandemic. The sample size in each category (0 to 3 assessments taking place during the pandemic) is possibly too low to document effects. It is noteworthy that a strong relationship between environmental as well as social barriers and difficulties in community participation has been found in the chronic phase following TBI by Kersey et al. [30]. Hence, the COVID-19-related restrictions may have contributed to the decline in overall participation and social participation during the study period. Venkatesan et al. did document a similar reduction in participation between TBI survivors and the general population during the COVID-19 pandemic [28]. However, there are also indications that COVID-19 may not restrict participation beyond the consequences of TBI itself [31].

Higher global functioning predicted higher levels of participation and productivity. This is in accordance with several previous studies assessing overall participation [10,32], but has particularly been well documented for return to work [33]. Furthermore, in the current study, higher education was positively related to participation level but did not influence the participation trajectory, as has been found previously [10,16].

In our study, younger age was predictive of higher participation, in line with previous findings of older age predicting lower levels of participation as well as progressively worsening participation over time [10,13]. Age, however, may naturally influence the level of participation in normal aging, with or without TBI, as both individuals with TBI and healthy controls have previously reported decreasing participation in leisure activities with normal aging [34]. Interestingly, Juengst et al. found that older adults in the chronic phase after moderate-to-severe TBI reported the highest participation satisfaction across life areas, despite having the lowest participation frequency [35]. These findings highlight that measuring the level of satisfaction in participation activities adds valuable information in addition to participation frequency.

The influence of being married or cohabitating on overall and social participation is expected as partner status is included in PART-O [36]. Our findings of the positive relationship between living with a partner and social participation may also be attributed to the psychosocial influence of cohabitating with a partner. Of note, Hart and colleagues found that being single predicted a decline in social participation [16]. Another study has also shown that having a history of TBI is associated with greater loneliness compared with individuals without TBI [37]. Indeed, cognitive symptoms such as deficits in executive functioning, memory, attention, social cognition, and psychomotor slowing, along with reduced global functioning, have a negative impact on participation and social functioning. The positive relationship between living with a partner and social participation may be explained by the psychosocial support received from the partner, both regarding initiating and planning social activities as well as during the participation in social activities.

Neither the goal-oriented intervention nor symptom burden influenced the trajectories of participation in the present study. A bit surprising was the lack of influence of symptom burden on participation in this cohort. In contrast, Kinney et al. [38] documented a clear association between post-concussion symptoms and participation, and several previous studies have found a negative association between depression and participation [10,15,39]. Possibly the functional aspects outweighed the influence of symptoms in the present cohort of individuals with TBI. The goal-oriented intervention focused on the individual problem areas, reduced symptoms, and improved overall health-related quality of life [17]. However, symptom burden was not associated with participation level or trajectories in the present study. The short time frame of one year may also be insufficient to detect subtle long-term changes in participation [4,21].

## 5. Limitations

This study did not include a measure of participation satisfaction, i.e., the Participation Assessment with Recombined Tools-Subjective (PART-S), which could possibly have added valuable information regarding the participants’ subjective appraisal of the participation activities. Furthermore, the study did not include TBI survivors at a specific time since injury; hence, temporary analysis in the light of specific time points in the course of injury could not be investigated, e.g., one, two, or three years post-injury. The follow-up was limited to one year, and the finding of a statistically significant decline in participation was small.

## 6. Conclusions

Participation is recognized as a primary goal of TBI rehabilitation. The results of this study provide important information regarding positive and negative factors influencing participation levels in the chronic phase following TBI. Understanding which factors increase the risk of poor participation as well as which factors have a positive impact on participation level may allow rehabilitation professionals to identify patients who are at risk of poor participation outcomes and to develop targeted interventions. Our study highlights that it may be worthwhile to have a particular focus on support and follow-up for persons living alone after TBI, as they may be less community-engaged or have less social support. Future studies should investigate the minimal clinically important difference of PART-O.

## Figures and Tables

**Table 1:** Demographic and clinical characteristics.

	Participants Total (*n* = 120)
*Sociodemographics*	
Age, mean (SD), years	45.15 (14.44)
Males, no. (%)	85 (71%)
Education level, mean (SD), years	13.17 (2.33)
Paid employment, no. (%)	60 (50%)
Married/domestic partner, no. (%)	68 (56.7%)
*Injury-related variables*	
Months post-injury, median (IQR Q1–Q3)	53 (44, 81)
Lowest unsedated GCS, median (IQR Q1–Q3)	9 (5, 14)
Severity of injury	
Mild TBI (GCS 13–15), no. (%)	41 (34%)
Moderate TBI (GCS 9–12), no. (%)	18 (15%)
Severe TBI (GCS 3–8), no. (%)	54 (45%)
Unknown severity, no. (%)	7 (6%)
Cause of injury	
Transport-related accident	50 (41.7%)
Fall	39 (32.5%)
Violent incident	9 (7.5%)
Other	18 (15%)
Unknown	4 (3.3%)
*Function*	
GOSE score, median (IQR Q1–Q3)	6 (5, 7)
BRIEF-A GEC score, median (IQR Q1–Q3)	55 (48, 62)
RPQ score, median (IQR Q1–Q3)	24 (13, 32)
PHQ-9 score, median (IQR Q1–Q3)	7 (4, 11)

Abbreviations: BRIEF-A = Behavior Rating Inventory of Executive Function—Adult version, GCS = Glasgow coma scale, GOSE = Glasgow Outcome Scale Extended, No = number of, IQR = interquartile range, PHQ-9 = Patient Health Questionnaire-9, RPQ = Rivermead Post Concussion Symptoms Questionnaire, SD = standard deviation, TBI = traumatic brain injury.

**Table 2:** Linear mixed-effects model analysis of PART-O Total score and the Productivity and Social subscales.

	PART-O Total Score			PART-O Productivity Subscale			PART-O Social Subscale		
Predictors	Coefficient	95% CI	*p*-Value	Coefficient	95% CI	*p*-Value	Coefficient	95% CI	*p*-Value
Constant	1.61	1.0 to 2.2	<0.001	1.37	0.50 to 2.24	<0.001	1.77	0.85 to 2.71	<0.001
Time	−0.01	−0.03 to −0.01	**0.035**	−0.01	−0.03 to 0.01	0.241	−0.02	−0.04 to 0.01	0.128
Age	−0.01	−0.01 to −0.01	**0.005**	−0.01	−0.01 to 0.01	0.132	−0.01	−0.02 to −0.01	**<0.001**
Female (vs. male)	0.06	−0.08 to 0.20	0.413	0.01	−0.20 to 0.21	0.964	0.16	−0.06 to 0.37	0.163
Married/Cohabitating	0.38	0.25 to 0.51	**<0.001**	0.14	−0.05 to 0.33	0.140	1.15	0.95 to 1.35	**<0.001**
Education	0.04	0.01 to 0.07	**0.014**	0.05	0.01 to 0.10	**0.014**	0.04	−0.01 to 0.09	0.079
TBI symptom burden (RPQ)	−0.01	−0.1 to 0.01	0.829	0.01	−0.01 to 0.01	0.223	0.01	−0.01 to 0.01	0.595
Executive dysfunction (BRIEF−A)	−0.01	−0.01 to 0.01	0.084	−0.02	−0.03 to −0.01	**0.004**	−0.01	−0.01 to 0.01	0.752
Depression (PHQ-9) *Global functioning*	0.11	−0.06 to 0.28	0.204	0.24	−0.01 to 0.48	0.051	0.01	−0.24 to 0.27	0.910
GOSE 6 vs. ≤5	0.30	0.14 to 0.46	**<0.001**	0.83	0.60 to 1.1	**<0.001**	0.17	−0.07 to 0.42	0.161
GOSE 7 vs. ≤5	0.51	0.32 to 0.69	**<0.001**	1.33	1.1 to 1.6	**<0.001**	0.28	−0.01 to 0.56	0.059
*Assessments during COVID*									
T3 during the pandemic	−0.07	−0.26 to 0.13	0.511	−0.39	−0.67 to −0.11	**0.006**	0.15	−0.15 to 0.44	0.332
T2 & T3 during the pandemic	−0.17	−0.36 to 0.02	0.083	−0.58	−0.85 to −0.31	**<0.001**	0.13	−0.15 to 0.42	0.362
T1, T2 & T3 during the pandemic	−0.11	−0.30 to 0.09	0.274	−0.46	−0.74 to −0.18	**0.001**	0.09	−0.20 to 0.39	0.538

Other results of the interaction of treatment group by time omitted due to non-significant results. Abbreviations: BRIEF-A = Behavior Rating Inventory of Executive Function—Adult version, CI = confidence interval, GOSE = Glasgow Outcome Scale Extended, PHQ-9 = Patient Health Questionnaire-9, RPQ = Rivermead Post Concussion Symptoms Questionnaire, TBI = traumatic brain injury.

## Data Availability

The data presented in this study are available on request from the corresponding author. The data are not publicly available due to national data protection rules.
